# The Change in Paradigm for NSCLC Patients with EML4–ALK Translocation

**DOI:** 10.3390/ijms23137322

**Published:** 2022-06-30

**Authors:** Alessandra Bearz, Elisa De Carlo, Alessandro Del Conte, Michele Spina, Valentina Da Ros, Elisa Bertoli, Alberto Revelant, Brigida Stanzione, Umberto Tirelli

**Affiliations:** 1 Dipartimento di Oncologia Medica, Centro di Riferimento Oncologico di Aviano (CRO) IRCCS, 33081 Aviano, Italy; elisa.decarlo@cro.it (E.D.C.); alessandro.delconte@cro.it (A.D.C.); mspina@cro.it (M.S.); vdaros@cro.it (V.D.R.); elisa.bertoli@cro.it (E.B.); brigida.stanzione@cro.it (B.S.); 2 Department of Medicine (DAME), University of Udine, 33100 Udine, Italy; 3 Dipartimento di Radioterapia, Centro di Riferimento Oncologico di Aviano (CRO) IRCCS, 33081 Aviano, Italy; alberto.revelant@cro.it; 4 Tirelli Medical Group, 33081 Pordenone, Italy; utirelli@tirellimedical.it

**Keywords:** NSCLC, ALK, targeted therapies

## Abstract

The severe prognosis linked with a lung cancer diagnosis has changed with the discovery of oncogenic molecularly driven subgroups and the use of tailored treatment. ALK-translocated advanced lung cancer is the most interesting model, having achieved the longest overall survival. Here, we report the most important paradigmatic shifts in the prognosis and treatment for this subgroup population occurred among lung cancer.

## 1. Introduction

Lung cancer is the world’s leading cause of cancer-related death [1]. The severity is due to the fact that roughly 75% of patients with non-small-cell lung cancer (NSCLC) are diagnosed with lung cancer when it has already metastasized or locally progressed; at that time, the 5-year survival rate is only 7%, although it is gradually improving [2,3]. Tobacco smoking is the leading cause of lung cancer, responsible for over 90% of lung cancer deaths in countries where smoking is prevalent in both sexes [4].

### Disease Biology

Historically lung cancer is divided into non-small-cell lung cancer, which is predominant, and small-cell lung cancer, accounting for roughly 13% overall; among non-small-cell lung cancer, adenocarcinoma is the most frequent histology [5]. Multiple molecular targets have been identified, mostly in lung adenocarcinomas, which may be targeted by tailored drugs. Multiple drugs targeting major oncogenic signaling pathways have been developed and have shown clinical activity; many of them have shown superiority in comparison with cytotoxic chemotherapy and have become first-line treatment. At present, it is important to check the presence of a mutated oncogenic pathway at the diagnosis of metastatic lung cancer to give the chance of tailored treatment, due to the impact it has on survival. Among the tyrosine kinase inhibitors (TKIs), ALK inhibitors have achieved the longest survival times in patients undergoing systemic treatment for advanced lung cancer [6,7]. The median overall survival (OS) exceeds 80 months for ALK-positive lung cancer [8]. The ALK-positive disease in NSCLC represents a unique model for having the longest overall survival achieved with systemic treatment, the available treatment sequencing, and the efficacy of systemic treatments over brain metastases.

**Figure 1:**
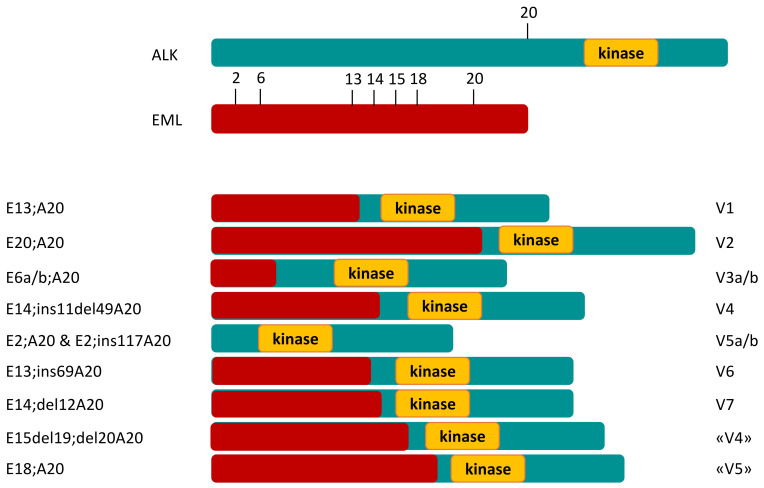
Alternative splicing leads to several variants of EML4–ALK fusion gene.

Constitutive expression of the ALK gene via fusion with another gene within chromosome number 2 can promote the growth of tumors with abnormal ALK expression [9].The echinodermic microtubule-associated protein-like-4 (EML4)–ALK fusion gene was discovered in NSCLC patients in 2007 [10]. EML4–ALK proteins are highly transforming and pathogenic in NSCLC due to increased oligomerization and constitutive, kinase-activating autophosphorylation: through activation of several downstream signaling pathways such as RAS/MAP kinase and PI3K/AKT, they lead to cell proliferation and de-differentiation. According to alternative splicing, several variants of EML4–ALK have been detected [11], as shown in Figure 1.

To date, the prognostic value of the variants is still not clear, due to the significant heterogeneity among the studies and different analytical methods: Common methods to detect *ALK* rearrangement are fluorescence in situ hybridization (FISH) and immunohistochemistry (IHC), which cannot identify the different *ALK* variants, while real-time polymerase chain reaction (RT-PCR) or next-generation sequencing (NGS) should be preferred to detect the different variants [12]. Epidemiological data suggest that ALK fusions occur in approximately 5% of NSCLC patients [13].

## 2. Historical Treatment of ALK-Positive NSCLC

The first ALK inhibitor was the tyrosine kinase inhibitor (TKI) crizotinib, a drug initially developed as a C-Met inhibitor and then commercialized as an ALK inhibitor [14]. The limits for crizotinib were the minor activity in the brain, owing to a poor drug penetration in the central nervous system (CNS), and the onset of resistance mutations under the pharmacological pressure of crizotinib. Subsequent next-generation TKIs have been designed in order to have a better penetration in the CNS and activity even against the resistance mutations; many of them (e.g., ceritinib, alectinib, brigatinib, ensartinib, and lorlatinib) have been tested in phase III, randomized trials in comparison with standard first-line treatment (chemotherapy or crizotinib) in advanced ALK-positive patients, and due to their demonstrated significative superiority, most of them have been approved by the Food and Drug Administration (FDA) and the European Medicine Agency (EMA) for the management of ALK-positive NSCLCs [15]. The current standard first-line therapy in advanced ALK-positive NSCLC is next-generation ALK inhibitors such as alectinib, brigatinib, or lorlatinib. Progression-free survival (PFS) with alectinib significantly exceeds that of crizotinib, with 34.8 months versus 10.9, respectively, in an updated analysis of the phase III trial ALEX [16]; in addition, progression-free survival with brigatinib has been demonstrated superior to that of crizotinib in advanced ALK inhibitor-naïve ALK-positive NSCLC patients, with 24.0 versus 11.0 months, respectively [17]. However, these treatments consistently lead to the accumulation of resistance, which facilitates disease progression. Optimal treatment options for patients following disease progression on second-generation ALK TKIs remain to be determined. Lorlatinib is a third-generation ALK inhibitor; in a phase II study, lorlatinib demonstrated a median PFS of 6.9 months among patients who had received one or more second-generation ALK TKIs [18]. Based on these results, lorlatinib was approved for second- or third-line treatment of ALK-positive NSCLC. Among the 139 patients treated in a global phase II trial, lorlatinib demonstrated significantly greater efficacy among patients with ALK resistance mutations compared with those without [19]. In a multicenter retrospective analysis, the chemotherapy based on platin and pemetrexed combination demonstrated modest clinical activity, with a median PFS of 4.3 months, suggesting that maybe ALK-positive tumors may be less sensitive to chemotherapy after they have become resistant to ALK TKIs [20]. In addition, lorlatinib in 2021 received approval for first-line treatment for ALK-positive lung cancer based on the results of the pivotal phase III Crown trial [21].

**Figure 2:**
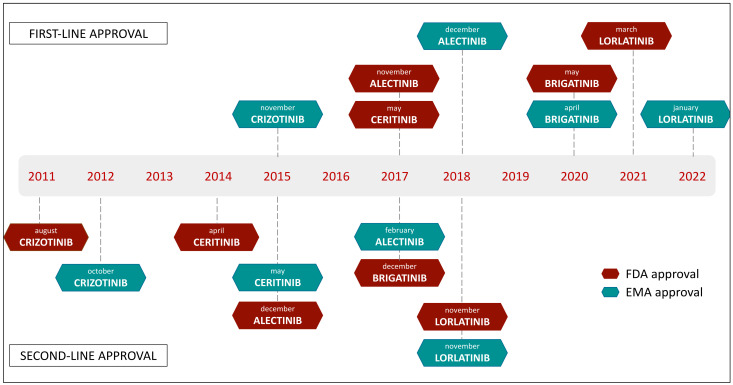
Regulative approval timelines of ALK TKIs, in first-line and second-line settings.

In Figure 2, we report the timeline of ALK TKIs’ approval by regulatory agencies.

In this review, we underline the change in paradigm introduced by the ALK inhibitors in the treatment of advanced lung cancer positive for ALK as follows:

- In advanced ALK-positive NSCLC, the long expectancy of survival makes the ALK model unique;
- The treatment of intracranial disease is available without radiotherapy, and the prediction of survival is irrespective of brain disease.

## 3. ALK Inhibitors and the Expectancy of Survival

For decades, the natural history of untreated advanced NSCLC has been considered to be poor, with a median survival between 2 and 5 months from diagnosis [22]. The 20-year history of the treatment of advanced NLSCLC was characterized by poor gains in terms of PFS, resulting in even minimal advances in overall survival (OS) until the discovery of the existence of oncogenic-driver mutations, making NSCLCs sensitive to tyrosine kinase inhibitors such as the ALK- or EGFR-positive NSCLCs. The systemic treatment for advanced NSCLC was established to include a platinum compound, together with paclitaxel, vinorelbine, or gemcitabine [23]; due to the introduction of new drugs in the first-line treatment setting and patient selection based on histological subtypes, the median survival for patients with advanced NSCLC receiving platinum-based chemotherapy in combination with agents targeting specific histologies and mutations has improved to 12 months or slightly longer in randomized controlled trial populations [24]. For non-squamous histology, the approval of a maintenance strategy had a minimal impact also on overall survival, with a median OS of 13.9 months [25]. Despite the availability of multiple treatment options in the second-line setting, clinical outcomes remain poor with minimal differences and poor impact adding second-line treatment [26]. Response rates are, on average, less than 10%, and median survival is 7 to 9 months from the start of second-line therapy [27]. The discovery of driver oncogenic mutations characterizing different lung cancers and resulting in different defined subgroups of lung cancer patients opened the era of targeted therapies with specific tyrosine kinase inhibitors [28]. Thus far, in the setting of advanced or metastatic NSCLC, it is of paramount importance to profile the tumor to test the existence of driver mutations eventually leading to a tailored treatment with an impact on overall survival. Potentially actionable mutations may be found in up to 64% of lung adenocarcinomas [29], although not all of these mutations have clinically validated treatments.

About 5% of NSCLC are driven by a gene mutation known as anaplastic lymphoma kinase. Targeted treatments for those with advanced *ALK-*mutated NSCLC have been developed and found to be more effective than chemotherapy. There are several ALK inhibitors already approved; the first-generation ALK inhibitor crizotinib, the first to be approved by the FDA for the treatment of ALK-positive patients; second-generation ALK inhibitors such as ceritinib, ensartinib, alectinib, and brigatinib; and lastly, the third-generation inhibitor lorlatinib. The Cochrane meta-analysis has reviewed 11 studies including 2874 participants: Among those, 6 studies compared an ALK inhibitor (crizotinib, ceritinib, or alectinib) to chemotherapy, and 5 studies compared a next-generation ALK inhibitor (alectinib, brigatinib, or lorlatinib) to crizotinib [30]. The meta-analysis confirmed the superiority of the treatment with ALK inhibitors in terms of PFS, regardless of line of treatment, in comparison with chemotherapy; ALK inhibitors slightly improved OS, despite most included studies having a significant number of participants crossing over from chemotherapy to receive an ALK inhibitor after the study period. ALK inhibitors increased objective response rate (ORR) including in patients with measurable baseline brain metastases when compared with chemotherapy [16,17,21,31,32,33].

Second- or third-generation ALK inhibitors resulted in a large increase in PFS, particularly in participants with baseline brain metastases, when compared with crizotinib. Second- or third-generation ALK inhibitors increased OS and ORR including a response in measurable brain metastases, compared with crizotinib, mostly in the first-line setting. These results justify the actual choice of first-line treatment for advanced ALK-positive NSCLC with a second- or third-generation ALK TKI.

### 3.1. First-Generation ALK Inhibitor

Crizotinib was the first ever-approved ALK inhibitor by the US Food and Drug Administration (FDA). Crizotinib is an oral tyrosine kinase inhibitor, which targets ALK, c-ros oncogene 1 (ROS-1), and c-MET. After striking results of phase I/II trials, the phase III PROFILE 1007 trial, comparing crizotinib with standard chemotherapy as second-line (either pemetrexed or docetaxel) treatment in patients previously treated with chemotherapy, demonstrated an improvement in ORR (65% vs. 20%, *p* < 0.001), median progression-free survival (7.7 months vs. 3.0, *p* < 0.001), and quality of life in favor of crizotinib [34]. The promising result of the PROFILE 1007 trial led to the phase III PROFILE 1014 clinical study, with the goal to assess the efficacy of the ALK inhibitor crizotinib compared with standard chemotherapy with pemetrexed plus platinum as the first-line treatment for metastatic ALK-positive NSCLC patients. In the first-line setting, crizotinib demonstrated an improved objective response rate (ORR) (74% vs. 45%, *p* < 0.001) and PFS (10.9 vs. 7.0 months, *p* < 0.001) [35]. The trial defined the role of crizotinib as the first-line standard of care. In both trials, in the first- and second-line settings, there was no overall survival difference, likely because of cross-over. However, resistance to crizotinib occurred after approximately a median of 8 months of treatment, due to a pharmacodynamic mechanism of resistance or a pharmacokinetic limit, with an inadequate penetration into the CNS via crizotinib [36]. The development of secondary resistance mutations in the kinase target, gene amplification of the primary oncogene, and upregulation of bypass signaling tracts are the main mechanisms of resistance to crizotinib; second- and third-generation ALK TKIs can overcome these mutations, and they have different levels of sensitivity to secondary mutations, according to in vitro assays [37]. The predisposition toward brain progression during crizotinib treatment is due to poor accumulation of the drug in the CNS, as a result of a substrate of the drug efflux pump p-glycoprotein [38,39]. Therefore, second- and third-generation ALK TKIs have been specifically designed to be able to reach a therapeutic dosage in the CNS to prevent new metastases and treat the ones already present.

### 3.2. Next-Generation ALK Inhibitors

Among the second-generation ALK TKIs, ceritinib is 20 times as potent as crizotinib, with activity and efficacy against ALK mutations arising after crizotinib exposure, particularly L1196M, G1269A, I1171T, and S1206Y. Ceritinib inhibits the autophosphorylation of ALK and shows activity against IGF-R1, IR, and ROS-1 [40]. Ceritinib has also the ability to penetrate the blood–brain barrier. In a phase III randomized multicenter ASCEND-4 trial, previously untreated ALK-positive NSCLC patients were randomized to receive ceritinib or platinum-based chemotherapy until disease progression or unacceptable toxicity, including those with asymptomatic or stable brain metastases. The results demonstrated an mPFS of 16.6 months with ceritinib versus 8.1 months with standard chemotherapy treatment (HR 0.55; 95% CI 0.42–0.73), and an ORR, respectively, of 73% vs. 27%.; intracranial activity with ceritinib and chemotherapy was 46.3% vs. 21.2%, respectively. The most common toxicities of ceritinib were gastrointestinal toxicities of any grade, including diarrhea (85%), nausea (69%), vomiting (66%), abdominal pain (25%), and liver function tests abnormalities (grade 3 or 4, more than 70%), other than anorexia and fatigue [32]. The side effects were mitigated by reducing the dosage to 450 mg/die [41]. However, the poor tolerability of ceritinib inhibited the widespread clinical use of the drug.

Alectinib is a highly potent second-generation ALK TKI and rearranged during transfection (RET) gene inhibitor. Due to its robust clinical efficacy and better safety profile, alectinib is actually one of the two first-line options for metastatic ALK-positive NSCLC. Alectinib is not a substrate of the p-glycoprotein efflux transporter, allowing it to effectively penetrate the blood–brain barrier [7]. Alectinib demonstrated high efficacy against several crizotinib-resistant mutations in ALK, along with L1196M, G1269A, C1156Y, F1174L, 1151Tins, and L1152R but not G1202R. The phase III J-ALEX study enrolled 207 Japanese patients, who were randomized to receive alectinib 300 mg orally twice daily or crizotinib 250 mg twice daily. After a median follow-up of 42.2 months in the alectinib arm and 42.4 months in the crizotinib arm, the mPFS rates were 34.1 months and 10.2 months (HR 0.37) with alectinib and crizotinib, respectively [6,42]. The second phase III randomized ALEX trial enrolled 303 previously untreated Caucasian patients affected by metastatic ALK-positive NSCLC and compared alectinib 600 mg orally twice daily with crizotinib in a first-line setting. Median PFS was longer with alectinib than with crizotinib (34.8 vs. 10.9 months, HR 0.50; 95% CI 0.36–0.70); the 12-month event-free survival was significantly longer with alectinib, 68.4% (95% CI, 61.0–75.9%) than with crizotinib, 48.7% (95% CI, 40.4–56.9%); ORRs were 82.9% and 75.5%, respectively (p = 0.09). The intracranial activity of alectinib has been consistently demonstrated across all trials. In the ALEX trial, time to CNS progression was significantly longer in the alectinib group than in the crizotinib group (HR 0.16, *p* < 0.001), allowing the possibility for many patients with CNS disease to be treated with TKIs alone without local therapy (surgery or radiation) [43]. Alectinib is tolerated much better than crizotinib, with grade 3-to-5 toxicities in approximately 40% of patients. The side-effect profile includes nausea (14%), diarrhea (12%), vomiting (7%), elevated bilirubin (15%), myalgias (16%), elevated creatine kinase levels, anemia (20%), and photosensitivity (5%).

Brigatinib is an oral, potent, and selective ALK and ROS1 tyrosine kinase inhibitor, approved by FDA and European Medicine Agency (EMA) for treating ALK-positive NSCLCs who experience disease progression on first-line crizotinib and, more recently, for untreated, naive patients.

Brigatinib (AP26113) is a dual ALK/EGFR inhibitor with potent preclinical activity against ALK mutants resistant to crizotinib and other ALK inhibitors, initially developed by ARIAD; brigatinib potently inhibits the in vitro kinase activity of ALK and several mutant variants tested, including G1202R, and inhibited several other mutant kinases, including ROS1, FLT3, and mutant variants of FLT3 (D835Y) and EGFR (L858R and T790M) [44].

The recommended dose of brigatinib is 90 mg orally once daily for the first 7 days of treatment, which, if tolerated, is followed by escalation to 180 mg once daily. The lead-in week of brigatinib should avoid the onset of early pulmonary events–namely, interstitial lung disease, occurring between 3% and 6%, more frequent when the starting dose was 180 mg daily [45].

In the phase II study ALTA, brigatinib was studied in crizotinib-refractory ALK fusion-positive NSCLC. Patients were randomized 1:1 to take either oral brigatinib 90 mg once daily (arm A) or 180 mg once daily with a 7-day lead-in at 90 mg (arm B), stratified by brain metastases and best response to crizotinib. The primary endpoint was investigator-assessed confirmed ORR. In the primary analysis of the ALTA trial, the ORRs were 9.2 months in arm A and 12.9 months in arm B, which was updated last year, with 16.7 months for arm B [46]. Median OS rates were 29.5 months in arm A and 34.1 months in arm B. Independent review committee (IRC)-assessed confirmed intracranial objective response rates (iORRs) in patients with measurable baseline CNS lesions were 50% in arm A and 67% in arm B, with a median duration of confirmed intracranial response of 9.4 months and 16.6 months, respectively. Therefore, in this trial, the activity of brigatinib after progression on crizotinib and its extensive activity over CNS metastases were confirmed.

Brigatinib, in the first-line setting, was studied in the ALTA-1L trial that randomized patients with ALK-positive NSCLC having no previous treatment with either brigatinib or crizotinib. Brigatinib was found to have superior efficacy to crizotinib, with better PFS and intracranial response. The estimated 12-month PFS was 67% (95% CI, 56–75%) for brigatinib, compared with 43% for crizotinib (95% CI, 32–53); HR for disease progression or death was 0.49 (95% CI, 0.33–0.74); *p* < 0.001. Intracranial response rates were significantly higher in the brigatinib group than in the crizotinib group, at 78% (95% CI, 52–94%) and 29% (95% CI, 11–52%), respectively [16]. Updated data from the ALTA-1L study showed blinded-IRC PFS of 24.0 (95% CI, 18.5–NR) months with brigatinib, compared with 11.0 (95% CI, 9.2–12.9) months with crizotinib. Intracranial PFS was significantly better in the brigatinib group compared to crizotinib, 24 (95% CI, 12.9–NR) months compared with 5.5 (95% CI, 3.7–7.5) months, respectively. In particular, brigatinib not only induces shrinkage of the existing lesions in the CNS, thus improving intracranial PFS in patients with brain metastasis at baseline, in comparison with crizotinib, but it even prevents the onset of new metastasis, improving intracranial PFS of patients without intracranial metastasis at baseline, in comparison with crizotinib [17].

Ensartinib was compared with crizotinib in a global phase III trial enrolling ALK-positive, advanced, previously untreated NSCLC patients. Ensartinib proved superior efficacy to crizotinib in both systemic and intracranial diseases [33].

The efficacy of lorlatinib was confirmed in a global phase II trial involving patients with ALK-positive advanced NSCLC [18]. In the several cohorts of treatment, patients may be treatment-naïve, in progression after crizotinib without chemotherapy, pretreated with chemotherapy and crizotinib, or having one, two, or three prior ALK TKIs other than crizotinib. The overall objective response (ORR) was high in all the cohorts as well as the intracranial responses; in particular, ORR was 40%, and median PFS was 6.9 months among the patients who had received one or more second-generation ALK TKIs.

In the first-line setting of the CROWN study, lorlatinib was compared with crizotinib in a treatment-naïve advanced NSCLC, ALK-positive population; although results are still preliminary, lorlatinib led to strong clinical benefits, with a percentage of patients alive without disease progression at 12 months being 78% (95% CI, 70 to 84) in the lorlatinib arm and 39% (95% CI, 30 to 48) in the crizotinib arm (HR 0.28; 95% CI, 0.19 to 0.41; *p* < 0.001). Objective response occurred in 76% (95% CI, 68 to 83) of the patients in the lorlatinib group and 58% (95% CI, 49 to 66) of those in the crizotinib group, with the highest rates of IC activity reported so far, 82% of activity in patients with brain metastases at baseline [34].

Within the limits of a cross-trial comparison, due to different patient populations, different median follow-ups, and different randomized trial designs for next-generation ALK TKIs compared with crizotinib in first-line settings, we have a scenario listed in Table 1.

From the data reported in Table 1, it is evident that the median progression-free survival rates of all four drugs are significantly superior to that of crizotinib. The four drugs have different toxicity profiles, as shown in Table 2.

Responses to ALK inhibitors are often not durable, and acquired resistance can occur as on-target or off-target alterations. Studies are underway to explore the mechanisms of resistance and optimal treatment options beyond progression [37].

Moreover, association with co-mutations such as TP53 mutations, which are considered poor prognostic biomarkers, as well as variants of ALK fusion, may contribute to the activity [47].

Thus far, we know that the activity of brigatinib is regardless of variants 1 and 3 and the presence of TP53 co-mutation [17], and the PFS reached with alectinib is regardless of the different variants 1, 2, or 3 [48].

Tissue or liquid re-biopsies at the time of disease progression, even though not required by the approval status of any ALK inhibitor, may help in allowing individualization and optimization of the therapy strategy. On-target mutation resistances may be overcome by the third-generation ALK inhibitor lorlatinib, while off-target resistances, for example, *MET*, *HER2*, or *KRAS* alterations, may be targeted through specific inhibitors available in clinical trials or by off-label drug administration. 

**Figure 3:**
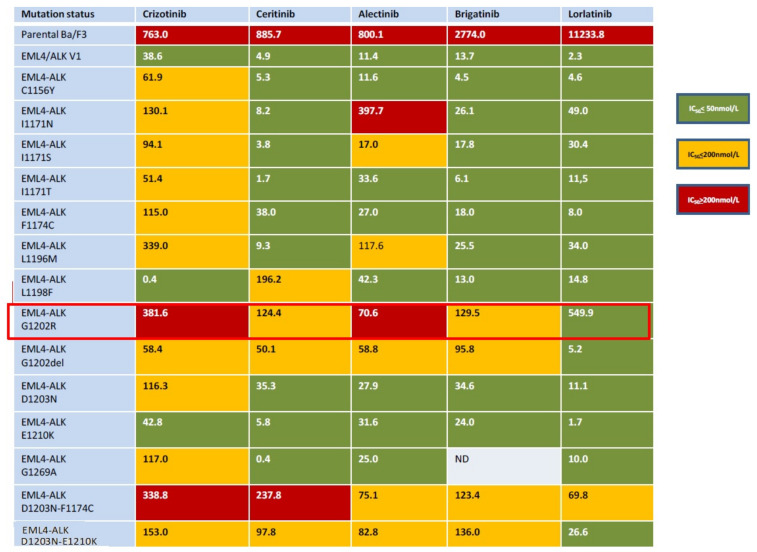
ALK inhibitors have different resistance profiles: highlighted in red is the efficacy of several ALK TKIs against the aggressive resistance mutation G1202R; in vitro data (modified from [37]).

In Figure 3, we report a chart showing the activity of several ALK TKIs on different ALK resistance mutations (modified with permission from [37]).

On the other hand, several patients failing TKI develop oligo-progression, which can be initially addressed with a local ablative strategy without switching to systemic therapy [49]. 

With all these studies, we are facing a new paradigm for ALK-positive patients in NSCLC. To date, the treatment for advanced ALK-positive NSCLC gives preference to an upfront use of second-generation ALK TKIs such as alectinib or brigatinib, due to longer PFS, longer IC PFS, and advantage over OS-although immature; the third-generation ALK TKI lorlatinib is used as a rescue, at systemic progression, leading to a sequencing strategy of treatment. However, third-generation ALK TKI lorlatinib may play a role as first-line treatment, as already approved by regulatory agencies, due to more robust efficacy data on the basis of cross-trial comparison, consistent with the strategy to “give the best first”.

Once available TKI options are exhausted, chemotherapy is available, although with modest clinical activity [20]. There are ongoing clinical trials regarding associations between VEGFR inhibitors and ALK TKIs, targeted therapies and ALK TKIS, and chemotherapy and ALK TKIs [50].

On the other hand, immunotherapy is not a recommended strategy for ALK-positive NSCLC patients, although PD-L1-positive rates among ALK-positive NSCLC patients are high, 46.7–50% and 13.3–16%, respectively [51]. Subgroup analyses of second-line immunotherapy trials have demonstrated that patients with NSCLC driven by *EGFR* or *ALK* mutations do not gain the same benefits from immunotherapy when given as a single agent [37]. The tumor microenvironment of ALK-positive NSCLC suggests a poorly immunogenic “immune desert” of ALK-positive NSCLC, preventing the successful use of immune checkpoint inhibitors [52]; most trials with immunotherapy in lung cancer have excluded patients with *ALK*-rearranged NSCLC, due to less efficacy of immunotherapy in this group [53].

With all these options, the strategy pathway for ALK-positive patients allows many months of treatment with sequential switches among the different ALK TKIs. Several articles from the literature, reporting retrospective data from real-world populations, underline robust overall survival, between 81 and 89.6 months, obtained through several switches among ALK TKIs and even chemotherapy [8,54,55,56]. Therefore, the once poor prognosis for lung cancer in the advanced stage has changed by the discovery of molecularly driven subgroups and by the use of targeted agents against the oncogenic mutation. The ALK translocation in NSCLC represents a model in oncology, having produced the longest OS in a once-a-time poor survival population. This is the first change in paradigm, showing that lung cancer is not poorly prognostic cancer anymore for everyone.

## 4. The Second Paradigmatic Shift in Lung Cancer: Management of Brain Metastases in ALK-Positive NSCLC

Historically, lung cancer was associated with poor prognosis, even worse in presence of brain metastasis at diagnosis, with overall survival ranging between 3 and 6 months [57]. Since the ALK population tends to develop brain metastasis relatively often, roughly 30% at diagnosis, and knowing that crizotinib levels in the brain are less than in the blood or in other tissues [58], further ALK TKIs—namely, second- and third-generation ALK TKIs—have been developed in order to pass the brain barrier and to be effective even in the sanctuary of the brain. The IC activity levels of brigatinib, ceritinib, alectinib, and lorlatinib have been demonstrated to be robust and consistent among the studies, inducing high quality of life and prolonged ORR. Whether brain radiotherapy should be offered up front, together with targeted therapies for oncogene-driven NSCLC with CNS involvement, has been a matter of controversy. A retrospective analysis of NSCLC patients with EGFR mutation and brain involvement published in 2017 demonstrated a survival advantage for the addition of cerebral irradiation to first-/second-generation EGFR inhibitors [59]: In this study, the rate of brain progression was slightly lower for patients treated with TKI and stereotactic (SRT) or whole-brain radiotherapy (WBRT) than for those treated with TKI alone, producing a significant benefit in terms of OS. However, the comparison with the brain activity of a newer EGFR TKI such as osimertinib points to a different possibility: With the bias of cross-trial comparison, in absolute terms, the rate of brain progression in all three patient groups (i.e., TKI-only; TKI and SRT; and TKI and WBRT) was much higher than that observed with osimertinib in the experimental arm of the Flaura study [60]. In ALK-positive disease, with an even higher incidence of brain involvement at diagnosis, and with the robust intracranial performance of newer TKIs such as alectinib, brigatinib, and lorlatinib, a similar strategy may be sustainable. A real-world retrospective analysis of ALK-positive advanced patients treated with alectinib showed efficacy comparable to that observed with TKI in registered clinical trials [61]. Overall, available data from the literature argue for a “radiation-free” first-line with second- and third-generation ALK TKIS, unless for patients diagnosed with brain metastases and life-threatening brain symptoms. For most of the ALK-positive NSCLC patients with brain involvement, surveillance using brain magnetic resonance imaging (MRI) is considered the preferable option; SRT may be offered at progression, even on the most potent, third-generation ALK TKI lorlatinib, while the use of the neurotoxic WBRT should be strongly discouraged for ALK-positive NSCLC, as long as potentially effective systemic treatments are still available [62].

**Figure 4:**
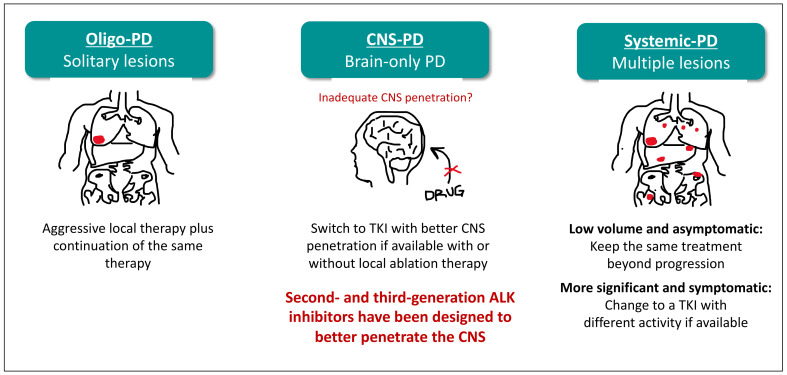
Different therapeutic strategies in the management of advanced ALK-positive NSCLC, according to pattern and site of progression.

In Figure 4, we report a diagram showing the decision-making key points among the different therapeutic strategies in the management of advanced ALK-positive NSCLC, according to pattern and site of progression.

For oligo-progressive disease, the ablative treatment may allow continued use of the same ALK inhibitor, without changing the treatment; however, if oligo-progression involves the brain, and if the first treatment is an ALK TKI with poor penetration of the brain, the shift to an ALK TKI with more brain activity should be encouraged. When the progression is in multiple sites, the systemic treatment should be changed.

The second change in paradigm for advanced lung cancer patients with ALK translocation is the recognition that prognosis is not influenced by brain metastasis, and the treatment up front should be a systemic, radiation-free treatment for most cases. Even at progression in the brain, radiotherapy can be spared, by shifting to ALK TKIs with more ability to pass the brain barrier.

## 5. Conclusions

Treatment of advanced lung cancer has dramatically changed in the last 25 years, and most of the improvement has dominantly been the result of the discovery of molecularly driven subgroups, which may be targeted with tailored treatments.

ALK-positive, advanced NSCLC gained so far the longest overall survival even with severe predictive and prognostic factors such as the presence of brain metastasis.

## Figures and Tables

**Table 1:** Updated efficacy data of next-generation ALK TKIs trials versus crizotinib.

Drug	Clinical Trial	# pts	CNS Mets at Baseline	ORR (%) (95% CI)	PFS (Months) in ITT (5% CI)	Intracranial Response Rate	Ref.
Lorlatinib	Crown	296	Lorlatinib: 26% Crizotinib: 27%	Lorlatinib: 76% (68–83) Crizotinib: 58% (49–66)	Lorlatinib: NE Crizotinib: 9.3 (7.6–11.1) HR:0.28 (0.18–0.41)	Lorlatinib 82% (57–96) Crizotinib: 23% (5–54)	[21]
Alectinib	ALEX	303	Alectinib: 38% Crizotinib: 42%	Alectinib: 82.9% (76.0–88.5) Crizotinib: 75.5% (67.6–822.1)	Alectinib: 34.8 (19.9–NE) Crizotinib: 10.4 (7.7–14.6) HR: 0.50 (0.36–0.70)	Alectinib: 81% (58–95) Crizotinib: 50% (28–72) HR: 0.32 (0.15–0.64)	[16]
Brigatinib	ALTA-1	275	Brigatinib: 29% Crizotinib: 30%	Brigatinib: 71% (62–78) Crizotinib: 60% (51–68)	Brigatinib: not reached Crizotinib: 9.8 (9.0–12.9) HR: 0.49 (0.33–0.74)	Brigatinib: 78% (52–94) Crizotinib: 29% (11–52)	[17]
Ensartinib	eXalt-3	290	Ensartinib: 33% Crizotinib: 39%	Ensartinib: 75% Crizotinib: 67%	Ensartinib: 25.8 Crizotinib: 12.7 HR: 0.51 (0.35–0.72)	Ensartinib: 64% Crizotinib: 21%	[33]

**Table 2:** Safety and toxicity profiles of next-generation ALK TKIs in trials compared with crizotinib.

Drug	Serious TRAEs	TRAES Leading to Dose Reduction (% pts)	TRAES Leading to Drug Discontinuation (% pts)	More Common TRAES	Ref.
Lorlatinib	Lorlatinib: 34% Crizotinib: 27%	Lorlatinib: 49% Crizotinib: 47%	Lorlatinib: 7% Crizotinib: 9%	Hypercholesterolemia Hypertriglyceridemia Weight increase Peripheral neuropathy Cognitive effects	[21]
Alectinib	Alectinib: 28% Crizotinib: 29%	Alectinib: 16% Crizotinib: 21%	Alectinib: 11% Crizotinib:13%	Anemia Myalgia Increased bilirubin Weight increase Musculoskeletal pain Photosensitivity	[16]
Brigatinib	Brigatinib: 28% Crizotinib: 29%	Brigatinib: 28% Crizotinib: 29%	Brigatinib: 12% Crizotinib: 9%	Increased CK Cough Hypertension Increased lipase Early-onset ILD	[17]
Ensartinib	Ensartinib: 24% Crizotinib: 20%	Ensartinib: 24% Crizotinib: 20%	Ensartinib: 9% Crizotinib: 7%	Rash Pruritus Pyrexia Increased transaminase	[33]
